# Impacts of Freshwater and Seawater Mixing on the Production and Decay of Virioplankton in a Subtropical Estuary

**DOI:** 10.1007/s00248-019-01362-2

**Published:** 2019-04-10

**Authors:** Wei Wei, Nannan Wang, Lanlan Cai, Chuanlun Zhang, Nianzhi Jiao, Rui Zhang

**Affiliations:** 1 College of the Environment and Ecology, Xiamen University, Xiamen, 361102 People’s Republic of China; 2 State Key Laboratory of Marine Environmental Science, Institute of Marine Microbes and Ecospheres, Xiamen University, Xiamen, 361102 People’s Republic of China; 3 College of Ocean and Earth Sciences, Xiamen University, Xiamen, 361102 People’s Republic of China; 4 Department of Ocean Science and Engineering, Southern University of Science and Technology, Shenzhen, 518055 People’s Republic of China

**Keywords:** Pearl River estuary, Freshwater–seawater cross-transplants, Viral production, Viral decay

## Abstract

Virioplankton is an important component of the aquatic ecosystem and plays multiple ecological and biogeochemical roles. Although the spatial and temporal distributions and dynamics of virioplankton have been well investigated in riverine and marine environments, little is known about the dynamics and environmental controlling mechanisms of virioplankton in estuaries. In this study, viral abundance, production and decay were examined in the Pearl River Estuary (PRE), one of the largest estuaries in China. The influences of freshwater and seawater mixing on viral ecological dynamics were evaluated with several cross-transplant experiments. In PRE, viral abundance, production and decay rates varied from 2.72 ± 0.09 to 27.5 ± 1.07 × 10^6^ viruses ml^−1^, 7.98 ± 2.33 to 16.27 ± 2.85% h^−1^ and 0.80 ± 0.23 to 3.74 ± 0.98% h^−1^, respectively. When the riverine and marine microbial community were transferred into simulated brackish water, viral production rates were markedly inhibited by 83.8% and 47.3%, respectively. The decay of riverine and marine virioplankton was inhibited by 21.1% and 34.2%, respectively, in simulated brackish water. These results indicate change of estuarine environmental factors significantly alters the dynamics of riverine and marine virioplankton. In addition, the effects of mixing on viral production and decay differed between high- and low-fluorescence viruses. High-fluorescence viruses seemed more resistant to decay than low-fluorescence viruses, whereas the production of marine low-fluorescence viruses seemed more resistant to inhibition than that of marine high-fluorescence viruses. Together, these results provide new insights into the ecological dynamics of virioplankton in estuarine environments.

**Electronic supplementary material:**

The online version of this article (10.1007/s00248-019-01362-2) contains supplementary material, which is available to authorized users.

## Introduction

Virioplanktons are the most abundant biological particles in the marine environment, with 10^5^ to 10^8^ particles ml^−1^ and are typically an order of magnitude more numerous than their hosts such as autotrophic and heterotrophic prokaryotes [1, 2]. During the past 20 years, it has become well known that viruses play important roles in marine microbial food webs [3–6]. Viruses can cause a high rate of microbial mortality by infection [2, 3], regulate elemental cycling via “viral shunt” [7, 8] and impact the structure of microbial communities by “killing the winner” [2, 9]. In addition, the elements of viral particles (e.g. carbon, nitrogen and phosphorus) released into the surrounding environment via decay have very important roles in local and global marine biogeochemical cycles [10, 11].

The dynamics of virioplankton are mainly regulated by two processes, i.e. production and decay [11]. The process of viral production entails the infection of a host cell by the virus and the subsequent release of progeny virus particles via lysis. Previous studies have shown that viral production is mainly related to the abundance and metabolic activity of their hosts [12–14]. In general, viral production in coastal regions and the surface layer is higher than that in open ocean and at depth [15, 16]. Regarding viral decay, the components of viruses are impacted by complex biotic and abiotic factors, e.g. solar radiation, temperature, adsorption to organic or inorganic particles, and extracellular enzymes and ultimately degrade and become part of the dissolved organic matter (DOM) [17–21]. Generally, the viral decay rate in eutrophic waters is higher than that in oligotrophic environments [22–25]. The balance of viral production and decay determines the biomass, population size and community composition of virioplankton and impacts virus–host interactions, thereby playing important roles in the microbial food web and biogeochemical cycles [3, 5, 6, 11].

An estuary is a special aquatic ecosystem that mainly consists of three regions: an inlet area, characterised as a freshwater environment; an offshore area dominated by seawater; and a brackish water region formed by the mixing of freshwater and seawater. Due to the dual effects of runoff and tidal flow, an estuary is a complex and multivariate aquatic environment, and it serves as a model ecosystem for studying the dynamics of microbial ecology and their environmental controls. Previous studies have shown that variations of temperature, salinity, organic carbon and nutrients in the estuarine environment impact the spatiotemporal distribution, activity, diversity and community structure of bacterioplankton (e.g. [26–28]). However, there are only a few studies on the ecological characteristics of virioplankton in the estuarine environment [29–33]. In particular, the factors impacting the population dynamics (such as production and decay) of estuarine virioplankton are unclear. Several questions remain unanswered; for example, how does the constant mixing of freshwater and seawater in an estuary affect the production and decay of viruses and therefore virus population size? Do marine and freshwater viruses show similar ecological behaviours at mixing areas? As virioplankton are important members of estuarine ecosystem, investigation of the above questions can improve our understanding of microbial food webs and biogeochemical cycling in estuaries.

Pearl River Estuary (PRE), the second largest estuary in China, is an important area of freshwater that flows into the South China Sea. The total area of PRE is ca. 2600 km^2^, and the average annual runoff is ca. 3200 × 10^8^ m^3^ a^−1^. A large volume of freshwater is transported from upstream rivers to the PRE annually. The intensive mixing of freshwater and seawater results in highly variable environmental conditions in the PRE, establishing clear environmental gradients of salinity, DOC, nutrient concentrations and other factors. In the PRE region, limited studies have shown that viral abundance is related to the dynamics of bacterioplankton, which are affected by environmental factors [34–36]. However, the effect of freshwater–seawater mixing with changes in environmental factors on viral population dynamics generally remains unknown. Therefore, the objectives of present study were (1) to investigate the ecological dynamics of virioplankton in representative areas of PRE and (2) to explore the effect of freshwater–seawater mixing on viral dynamics through a cross-transplant experiment.

## Materials and Methods

### Study Site and Sampling

**Fig. 1:**
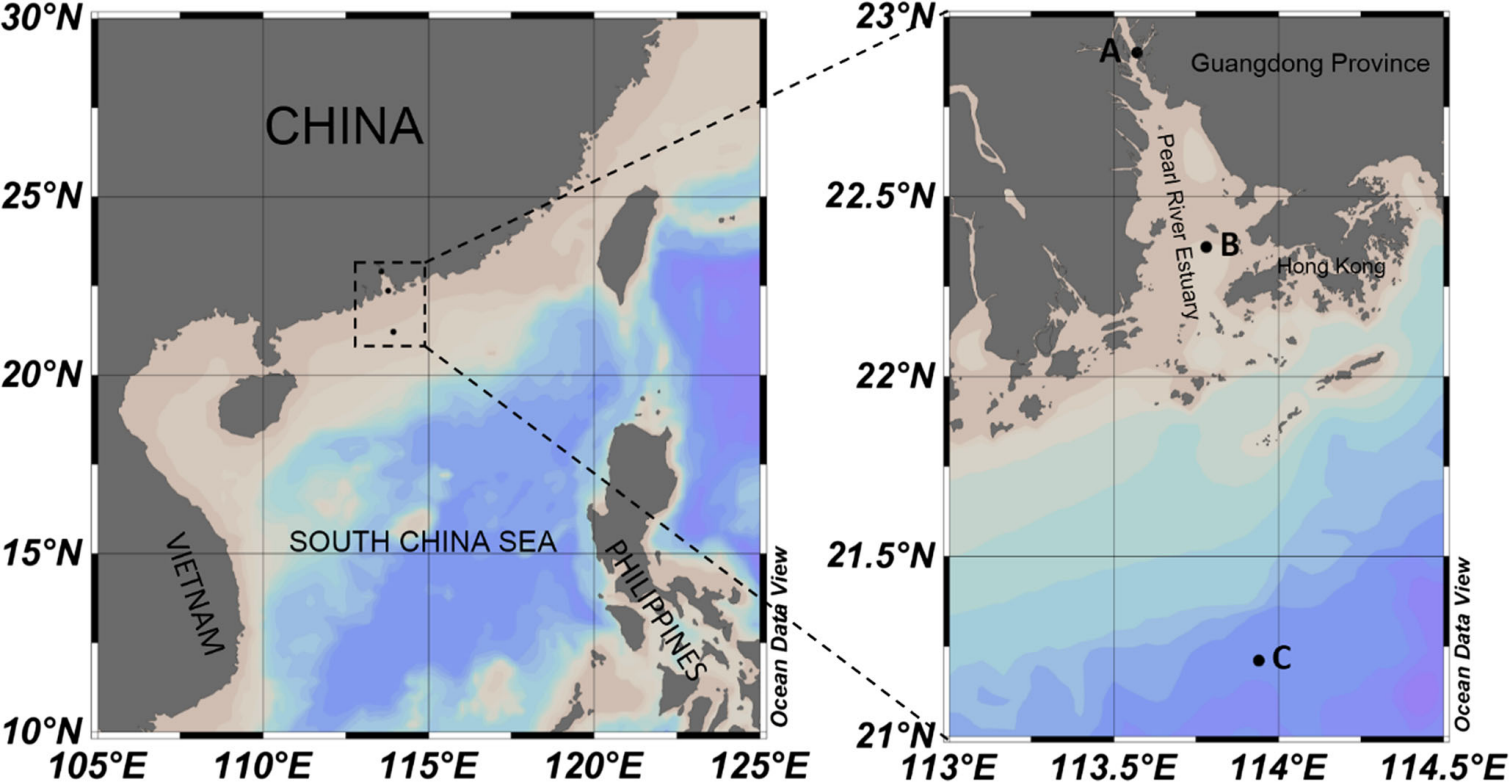
Map of the sampling stations in the Pearl River Estuary, South China Sea, China. The map was generated using Ocean Data View 4 software (https://odv.awi.de/)

Three representative stations were sampled in the PRE in the summer (July): one in a freshwater region (station A: 22.8° N, 113.6° E), one in a brackish water region (station B: 22.3° N, 113.7° E) and one in seawater region (station C: 21.2° N, 113.9° E) (Fig. 1). At each station, water samples were collected with acid-cleaned polycarbonate bottles from the surface (ca. 1 m depth) and then pre-filtered with 20 μm mesh filters to remove large particles and zooplankton. The pre-filtered water was used for determinations of picoplankton and virioplankton abundances and the subsequent viral production and decay experiment. A multi-parameter controller (U-50, Horiba, Japan) was used to obtain environmental data on temperature, salinity, turbidity, pH, dissolved oxygen (DO) and oxidation–reduction potential (ORP) at each station.

### Determination of Picoplankton and Virioplankton Abundances

Subsamples (2 ml) were fixed with glutaraldehyde (0.5% final concentration), incubated at 4 °C for 15 min in the dark, flash frozen in liquid nitrogen and then stored at − 80 °C [37]. With scatter diagrams of side scatter vs. red fluorescence and orange fluorescence vs. red fluorescence, the abundances of picoeukaryotes, *Synechococcus* and *Prochlorococcus* were directly determined by flow cytometry (Epics Altra II, Beckman Coulter, USA) [38, 39]. The samples for heterotrophic bacterial counting were stained with 1.0 × 10^−4^ SYBR Green I (*v*/*v*, final concentration, Molecular Probes), incubated for 15 min in the dark and analysed by flow cytometry with scatter diagrams of side scatter vs. green fluorescence and red fluorescence vs. green fluorescence [38, 39]. To obtain viral abundance, the samples were diluted with Tris–EDTA buffer (pH 8.0; Sigma), stained with 5.0 × 10^−5^ SYBR Green I (*v*/*v*, final concentration, Molecular Probes) and then incubated at 80 °C for 10 min in a thermostat water bath (DKB-501A, Shanghai Jinghong, China); abundance was then determined by flow cytometry [40, 41]. Data analyses were performed with FCS Express V3 software (De Novo Software, http://www.denovosoftware.com/).

### Lytic Viral Production Experiment

Lytic viral production rates were determined by using a reduction and reoccurrence assay according to Winget et al. [42] and Weinbauer et al. [43]. The 20-μm mesh and 3-μm filter pre-filtered samples (600 ml) were filtered by tangential flow filtration (TFF) with a 0.22-μm PVDF cartridge (Labscale, Millipore) to obtain the microbial concentrate and filtrate. Then, the filtrate was further ultrafiltered by 30 KD polysulfone cartridge (Labscale, Millipore) and TFF to obtain the viral concentrate and virus-free filtrate. Finally, the microbial concentrate (50 ml) and virus-free water (250 ml) were mixed, and 150 ml samples were incubated in 50-ml aseptic tubes (in triplicate) (Sigma, USA) at in situ temperature in dry bath incubators (MK-20, Hangzhou Allsheng, China) under dark conditions [16].

Subsamples (1 ml) were collected at time 0 and every 3 h over 12 h of incubation. The abundances of viruses were detected at each time point by flow cytometry (Fig. S1). The lytic viral production rate (viruses ml^−1^ h^−1^) was estimated by the viral accumulation in each 12 h incubation (e.g. Fig. S2) [44] and was calculated using the VIPCAL (http://www.univie.ac.at/nuhag-php/vipcal/) program [45].

The amount of prokaryotic death (ΔPA) was determined from the reduction of prokaryotic abundance in the viral production experiment. The formula is as follows:

$$ \Delta \mathrm{PA}=\left[\left({\mathrm{PA}}_{\max\ 1}-{\mathrm{PA}}_{\min\ 1}\right)/\left({t}_{\min\ 1}-{t}_{\max\ 1}\right)+\cdots +\left({\mathrm{PA}}_{\max\ n}-{\mathrm{PA}}_{\min\ n}\right)/\left({t}_{\min\ n}-{t}_{\max\ n}\right)\right]/n $$

where PA_max *n*_ and PA_min *n*_ represent the maximum and minimum prokaryotic abundance, respectively, at the *n*th peak of the prokaryotic abundance curve in the viral production experiment, and *t*_max *n*_ and *t*_min *n*_ are the corresponding culture times.

Then, the burst size (BS) is defined as follows:

$$ \mathrm{BS}=\mathrm{VP}\ \left(\mathrm{viruses}\ {\mathrm{ml}}^{\hbox{--} 1}\ {\mathrm{h}}^{\hbox{--} 1}\right)/\Delta \mathrm{PA} $$

The virus-mediated mortality of prokaryotic bacteria (VMM) is defined as follows:

$$ \mathrm{VMM}=\Delta \mathrm{PA}/{\mathrm{PA}}_0 $$

where PA_0_ represents the prokaryotic abundance at 0 h in the viral production experiment.

The carbon released by lysis (CRL) is defined as follows:

$$ \mathrm{CRL}=\Delta \mathrm{PA}\times 12.4\ \mathrm{fg} $$

where 12.4 fg is the carbon content of an average marine prokaryotic bacterium [46].

### Viral Decay Experiment

The virioplankton decay rates were determined with the method of Noble and Furhman [25]. The 20-μm and 3-μm pre-filtered water samples were filtered through 0.22-μm pore size polycarbonate filters to exclude the microbes, protozoa, and large particulate matters. The filtered water (150 ml) was then dispensed into 50-ml aseptic tubes (in triplicate) (Sigma, USA) and incubated in dry bath incubators (MK-20, Hangzhou Allsheng, China) at in situ temperature in the dark.

Subsamples (1 ml) were collected every 3 h for 12 h, and the viral abundances were determined at each time point by flow cytometry with the method described above (Fig. S3). The viral decay rate was calculated as the slope of the linear fitted curve of the viral abundance (ln-transformed) decline over the 12 h incubation (e.g. Fig. S4). Multiplying the slope by 100, the decay rate was expressed as a percentage per hour [25].

### Experimental Setup of the Freshwater–Seawater Cross-transplants

**Fig. 2:**
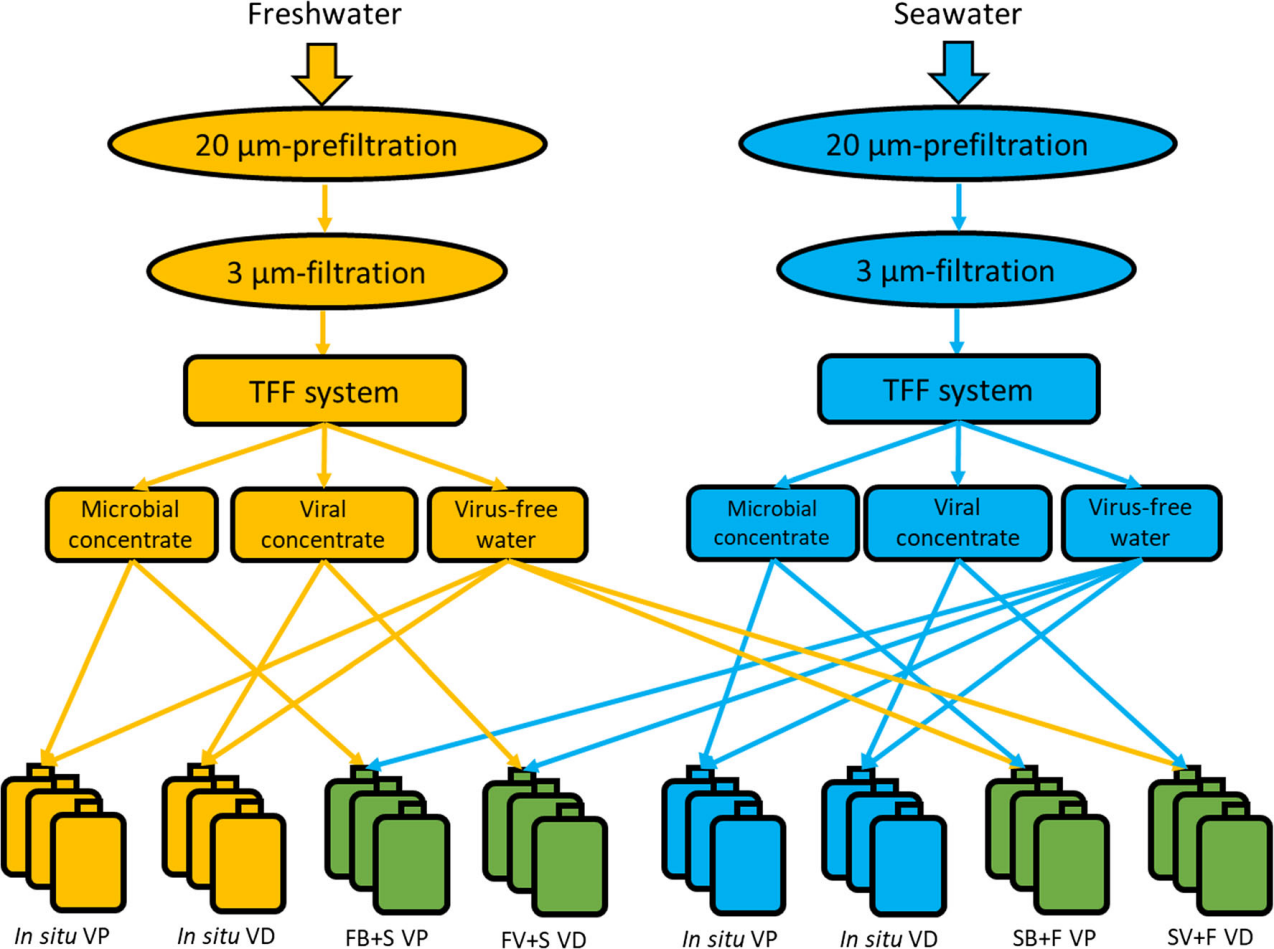
Schematics of freshwater–seawater cross-transplants for viral production and decay experiments based on TFF systems. Abbreviations represent tangential flow filtration (TFF), the treatment of riverine microbial community + virus-free seawater (FB + S), the treatment of riverine viral community + virus-free seawater (FV + S), the treatment of marine microbial community + virus-free freshwater (SB + F), the treatment of marine viral community + virus-free freshwater (SV + F), viral production experiment (VP) and viral decay experiment (VD)

As described above, using the TFF system, we obtained the riverine microbial concentrate, riverine viral concentrate and virus-free freshwater for station A and the marine microbial concentrate, marine viral concentrate and virus-free seawater for station C. After preparation of these six components, four treatments were applied in this experiment (Fig. 2): (1) riverine microbial community + virus-free seawater (FB + S), (2) riverine viral community + virus-free seawater (FV + S), (3) marine microbial community + virus-free freshwater (SB + F) and (4) marine viral community + virus-free freshwater (SV + F). TFF was widely used in microbial oceanography to obtain bacterial concentrate, viral concentrate and virus-free waters for dilution experiments such as measurement of viral production, viral decay, grazing, etc. [25, 43]. The manipulation influences of TFF on physiological and ecological activity of microorganisms, especially bacterioplankton and virioplankton, were considered to be insignificant [43]. In addition, in order to test whether sample handling/manipulation impact viral decay, we performed experiments with traditional vacuum filtration method [25] and compared to our TFF method. Statistical analysis indicated that there was no significant difference among filtration methods (Fig. S5). To develop a similar environmental condition (e.g. temperature, salinity and pH) as station B in the freshwater–seawater cross-transplants experiments, the volume of each component of the mixture was calculated according to Table S1. Each sample was mixed in a 50-ml aseptic tube (in triplicate) and incubated in a dry bath incubator at the in situ temperature of station B in the dark for 12 h to determine the viral production and decay rates.

### Statistical Analysis

In this study, the two-tailed Student's *t*-test was used to determine the statistical significance of each parameter with SPSS 19 software (SPSS Inc., Chicago, IL, USA), and differences were considered significant at a *p* value < 0.05.

## Results

### Environmental Parameters and Microbial Abundance

Generally, clear environmental gradients were observed from stations A to C, which is typical for estuarine areas (Fig. 1). The salinity increased significantly from stations A (0.14) to C (36.06), as did pH and DO. In contrast, the turbidity and ORP generally showed the opposite pattern of salinity (Table S1). The lowest temperature was recorded at station B, where the horizontal mixing of freshwater and seawater may have induced the vertical mixing of cold bottom water with warm surface water.

As the dominant picoplankton group in the PRE, heterotrophic bacteria showed a mean abundance of 2.26 ± 1.82 × 10^6^ cells ml^−1^, with abundance markedly decreasing along the estuary A–C axis from 4.33 ± 0.11 × 10^6^ cells ml^−1^ (station A) to 0.88 ± 0.09 × 10^6^ cells ml^−1^ (station C). In contrast, the *Synechococcus* and *Prochlorococcus* abundances increased from the upstream region, with 4.04 ± 0.10 × 10^4^ cells ml^−1^ and un-detected, respectively, to the downstream region, with 13.9 ± 0.92 × 10^4^ cells ml^−1^ and 16.1 ± 0.42 × 10^4^ cells ml^−1^, respectively. Picoeukaryotes had a lower abundance than did either *Synechococcus* or *Prochlorococcus* with the highest abundance of 8.97 ± 0.58 × 10^3^ cells ml^−1^ observed in the upper estuary. Their spatial distribution was similar to that of the heterotrophic bacterioplankton. Viruses displayed trends different from those of bacterioplankton. The maximum viral abundance was obtained for freshwater (station A), at 27.5 ± 1.07 × 10^6^ viruses ml^−1^, whereas the minimum value was observed for brackish water (station B) and was ca. one order of magnitude lower than the maximum value (2.72 ± 0.09 × 10^6^ viruses ml^−1^). The abundances of high- and low-fluorescence viruses presented the same trends as total virus abundance (Fig. S6) and were highest at station A, at 2.25 ± 0.15 × 10^6^ viruses ml^−1^ and 25.3 ± 1.25 × 10^6^ viruses ml^−1^, respectively, and lowest at station B, with 0.40 ± 0.12 × 10^6^ viruses ml^−1^ and 2.33 ± 0.08 × 10^6^ viruses ml^−1^, respectively.

### Spatial Variations of Lytic Viral Production and Viral Decay Rate

In the PRE, the mean lytic viral production rate was 12.24 ± 4.15% h^−1^ (1.05 ± 0.58 × 10^6^ viruses ml^−1^ h^−1^), and the rate increased from 7.98 ± 2.33% h^−1^ (2.19 ± 0.09 × 10^6^ viruses ml^−1^ h^−1^) at station A to 16.27 ± 2.85% h^−1^ (0.62 ± 0.03 × 10^6^ viruses ml^−1^ h^−1^) at station C (Table 2). Because the low-fluorescence viruses were the main subgroup of virioplankton, their production rates followed the same trend as that of total viruses and ranged from 7.39 ± 2.34% h^−1^ (1.87 ± 0.09 × 10^6^ viruses ml^−1^ h^−1^) to 12.47 ± 4.58% h^−1^ (0.35 ± 0.02 × 10^6^ viruses ml^−1^ h^−1^). The production rates of high-fluorescence viruses were much higher than those of low-fluorescence viruses (e.g. ca. 1.3-fold higher at station A, 2.3-fold higher at station B and 2.0-fold higher at station C). Moreover, the spatial distribution also differed between the high- and low-fluorescence viruses. Although the minimum of high-fluorescence viral production rate was shown in freshwater to be the same as low-fluorescence viral production rate, its maximum was observed in brackish water, with a value of 27.95 ± 4.66% h^−1^ (0.11 ± 0.03 × 10^6^ viruses ml^−1^ h^−1^), rather than in seawater.

The viral decay rates presented a significant difference in spatial distribution vs. viral production rates (Table 2). The highest viral decay rate of 3.74 ± 0.98% h^−1^ was obtained in freshwater, and the lowest value of 0.80 ± 0.23% h^−1^ was observed in brackish water. The decay rates of high- and low-fluorescence viruses both followed the pattern of the decay rate of total viruses, with peak values of 4.61 ± 0.83% h^−1^ and 3.46 ± 1.06% h^−1^, respectively, detected in freshwater. The decay rates of high-fluorescence viruses were all higher than those of low-fluorescence viruses, especially in brackish water, with the former subgroup having a ca. 4.5-fold higher decay rate.

### Viral Production and Decay Rates in the Experiment of Freshwater–Seawater Cross-transplants

In order to further study the effect of natural water mixing in the estuarine environment, i.e. the flow of upstream riverine freshwater and the return of downstream marine seawater into the estuary, on viral dynamics, an experiment with freshwater–seawater cross-transplants was performed.

**Fig. 3:**
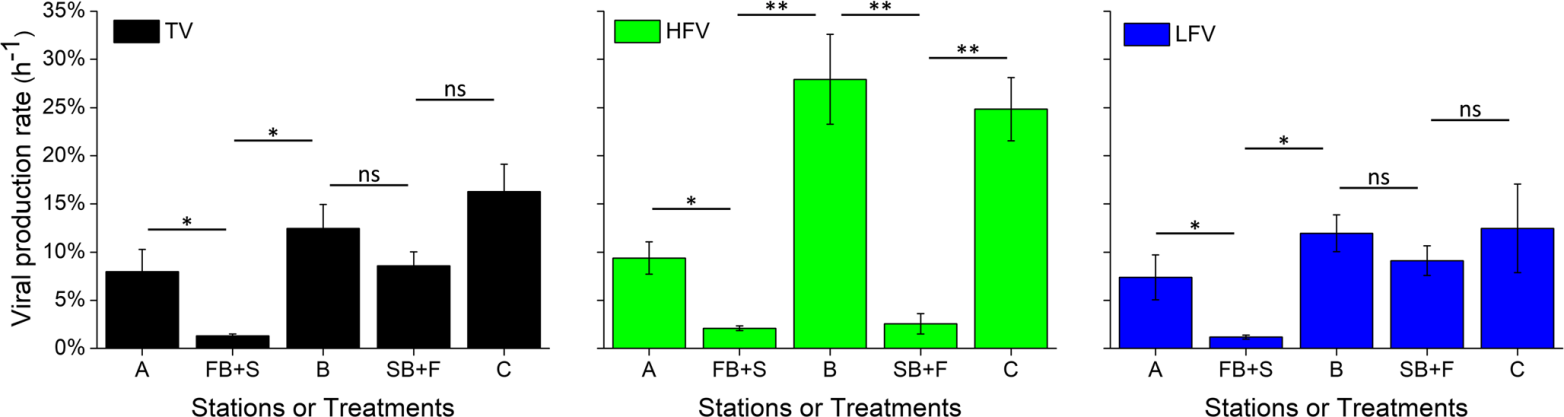
The total virus (TV), high-fluorescence virus (HFV) and low-fluorescence virus (LFV) production rates in a cross-transplant experiment in PRE. Error bars are the standard errors of triplicate measurements. ns no significant difference; *P ˂ 0.05; **P ˂ 0.01

When freshwater microbes were placed in seawater (FB + S treatment), the viral production rate was reduced from 7.98 ± 2.33% h^−1^ (station A) to 1.29 ± 0.20% h^−1^ (FB + S) (Fig. 3). Similarly, when seawater microbes were placed in freshwater (SB + F treatment), the viral production rate was reduced from 16.3 ± 2.85% h^−1^ (station C) to 8.59 ± 1.46% h^−1^ (SB + F). These results indicated that the production rates of both riverine and marine viruses were inhibited by freshwater–seawater mixing. Furthermore, the production rates of the two viral subgroups followed the same pattern as the production rate of the total viruses. In the FB + S treatment, the production rate was reduced from 9.40 ± 1.68% h^−1^ (station A) to 2.09 ± 0.24% h^−1^ for high-fluorescence viruses and from 7.39 ± 2.34% h^−1^ (station A) to 1.18 ± 0.21% h^−1^ for low-fluorescence viruses. Compared with the high- and low-fluorescence viral production rates 24.9 ± 3.29 h^−1^ and 12.5 ± 4.58 h^−1^, respectively, at station C, the production rates in the SB + F treatment were lower at 2.56 ± 1.06% h^−1^ and 9.13 ± 1.54%, respectively.

These results suggested the production rates of the total riverine viruses were inhibited to a greater extent than were those of the marine viruses under freshwater–seawater mixing. However, the trends were opposite between the high- and low-fluorescence viruses. As observed for the total viruses, the low-fluorescence viruses showed greater inhibition (i.e. $\frac{{\mathrm{VP}}_{\mathrm{A}}\hbox{--} {\mathrm{VP}}_{\mathrm{FB}+\mathrm{S}}}{{\mathrm{VP}}_{\mathrm{A}}}$, 84.0%, *P* < 0.05) of the freshwater microbes added into seawater than of the seawater microbes added into freshwater (i.e. $\frac{{\mathrm{VP}}_{\mathrm{C}}\hbox{--} {\mathrm{VP}}_{\mathrm{SB}+\mathrm{F}}}{{\mathrm{VP}}_{\mathrm{C}}}$, 26.8%). In contrast, the high-fluorescence viruses showed weaker inhibition under the FB + S treatment (77.7%, *P* < 0.05) than under the SB + F treatment (89.7%, *P* < 0.01), suggesting that the effect of freshwater–seawater mixing on production rate differed between high- and low-fluorescence viruses.

In order to test the effect of brackish water on production rates of indigenous, riverine and marine viruses, the environmental conditions of the FB + S and SB + F treatments were both artificially adjusted to match those of the station B treatment (brackish water). Viral production rate significantly differed among the three treatments, being highest at station B (12.48 ± 2.46% h^−1^), intermediate in the SB + F treatment (8.59 ± 1.46% h^−1^) and lowest in the FB + S treatment (1.29 ± 0.20% h^−1^). The same trend also appeared when separately examining the high- and low-fluorescence viruses, with rates of 27.95 ± 4.66% h^−1^ and 11.98 ± 1.92% h^−1^, respectively, at station B; 2.56 ± 1.06% h^−1^ and 9.13 ± 1.54% h^−1^, respectively, in the SB + F treatment; and 2.09 ± 0.24% h^−1^ and 1.18 ± 0.21% h^−1^, respectively, in the FB + S treatment. The results indicated that the simulated brackish water had different effects on production among the indigenous, riverine and marine viruses.

**Fig. 4:**
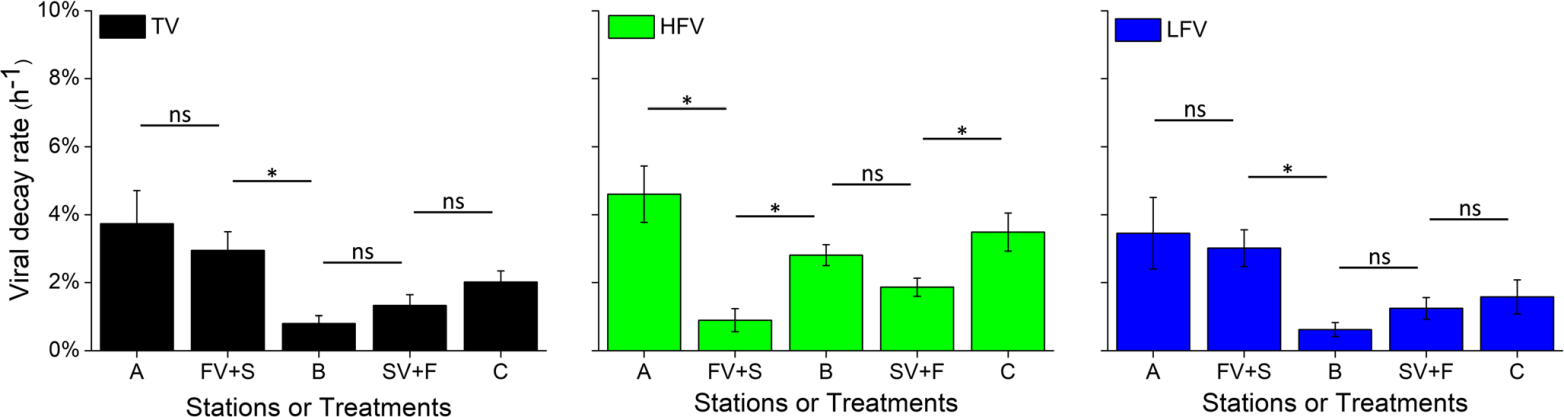
The total virus (TV), high-fluorescence virus (HFV) and low-fluorescence virus (LFV) decay rates in a cross-transplant experiment in PRE. Error bars are the standard errors of triplicate measurements. ns no significant difference; *P ˂ 0.05; **P ˂ 0.01

The effects of the freshwater–seawater cross-transplants on viral decay rate presented a pattern similar to that of their effects on viral production. Regardless of whether freshwater viruses were placed in seawater (FV + S treatment) or seawater viruses were placed in freshwater (SV + F treatment), viral decay rate declined, decreasing from 3.74 ± 0.98% h^−1^ (station A) to 2.95 ± 0.55% h^−1^ (FV + S) and from 2.02 ± 0.33% h^−1^ (station C) to 1.33 ± 0.31% h^−1^ (SV + F), respectively (Fig. 4). The decay rates of high- and low-fluorescence viruses showed the same trends as that of total viruses, indicating that the viral decay rates of total viruses and high- and low-fluorescence viruses were all inhibited by freshwater–seawater mixing.

Furthermore, the viral decay rate was reduced 21.1% for freshwater viruses placed in seawater and 34.2% for seawater viruses placed in freshwater, suggesting the viral decay of riverine viruses was inhibited slightly less than that of marine viruses under freshwater–seawater mixing. The decay rate of low-fluorescence viruses followed a similar pattern, with an inhibition of 12.8% under the FV + S treatment and 21.1% under the SV + F treatment. However, the decay rate of high-fluorescence viruses showed the opposite pattern, with stronger inhibition under the FV + S treatment (80.5%, *P* < 0.05) than under the SV + F treatment (46.6%, *P* < 0.05).

The environmental conditions of FV + S and SV + F were similar to those of station B, and the results revealed significant differences in decay rate among indigenous, riverine and marine viruses. The viral decay rates, from large to small, were 2.95 ± 0.55% h^−1^ under the FV + S treatment, 1.33 ± 0.33% h^−1^ under the SV + F treatment and 0.80 ± 0.23% h^−1^ at station B. These findings indicated that the simulated brackish water differentially affected the decay rates of indigenous, marine and riverine viruses. In addition, decay rate exhibited a different pattern between high- and low-fluorescence viruses. The highest decay rate of high-fluorescence viruses was detected in indigenous viruses (2.81 ± 0.31% h^−1^), whereas the lowest value for low-fluorescence viruses was found in indigenous viruses (0.62 ± 0.21% h^−1^), indicating that the simulated brackish water differentially affected decay between high- and low-fluorescence viruses.

## Discussion

In PRE, the heterotrophic bacterial, *Synechococcus* and picoeukaryotic abundances decreased with salinity in this investigation. This finding is consistent with previous studies in PRE and other tropical or subtropical estuaries [29, 32, 47, 48], indicating that salinity and inorganic and organic nutrients may be the dominant factors influencing the spatial distribution of picoplankton population size. In contrast, *Prochlorococcus* abundance increased with salinity, reflecting that the main habitat of *Prochlorococcus* is oceanic waters [47, 49].

The viral abundance, production rate and decay rate in PRE are similar to those reported in previous studies of estuarine environments (e.g. [15, 35]). Different from the oligotrophic marine environment, an estuary receives large amounts of organic nutrients, which increase microbial abundance and enhance metabolic activity [20, 50]. Thus, higher viral abundance and production and decay rates were observed in PRE compared with those reported for the open ocean. These findings indicate that viruses are highly dynamic and play important roles in the microbial ecology of the estuarine ecosystem [28, 35]. However, the measurement of in situ viral parameters cannot reveal the underlying mechanism of their dynamics because of the mixing of different sources of the microbial and viral community (freshwater and seawater) in estuarine areas. For instance, compared to the continuous trend (increasing or decreasing) of picoplankton abundance, viral abundance and decay showed their minimum values in the brackish water area (station B) (Tables 1 and 2). This finding suggested that the underlying mechanisms controlling the dynamics of virioplankton and bacterioplankton differed among the different regimes of the estuary. Consequently, VPR, which is usually used as an indicator of virus–host interactions, varied in relatively small geographic scale. This suggested a loose coupling between virioplankton and their potential autotrophic and heterotrophic hosts in the PRE, probably due to the fact that viruses from river water are mixing with prokaryotes from marine water and vice versa. To further study the influential mechanism of the estuarine environment on viral particles and related viral process parameters, freshwater–seawater cross-transplants were performed in this study.

**Table 1:** Microbial abundances at the three stations in PRE

Station	Het Bac (× 10^6^ cells ml^−1^)	Syn (× 10^4^ cells ml^−1^)	Pro (× 10^4^ cells ml^−1^)	Euk (× 10^3^ cells ml^−1^)	TV (× 10^6^ viruses ml^−1^)	HFV (× 10^6^ viruses ml^−1^)	LFV (× 10^6^ viruses ml^−1^)
A	4.33 ± 0.11	4.04 ± 0.10	ND	8.97 ± 0.58	27.5 ± 1.07	2.25 ± 0.15	25.3 ± 1.25
B	1.58 ± 0.24	8.73 ± 0.15	0.97 ± 0.05	3.98 ± 0.12	2.72 ± 0.09	0.40 ± 0.12	2.33 ± 0.08
C	0.88 ± 0.09	13.9 ± 0.92	16.1 ± 0.42	2.13 ± 0.17	3.83 ± 0.20	1.00 ± 0.06	2.83 ± 0.14

*Het Bac* heterotrophic bacterial abundance, *Syn Synechococcus* abundance, *Pro Prochlorococcus* abundance, *Euk* picoeukaryotic abundance, *TV* total virus abundance, *HFV* abundances of high-fluorescence viruses, *LFV* abundances of low-fluorescence viruses, *ND* not detected

**Table 2:** Viral parameters at the three stations in PRE

Station	TVP (h^−1^)	HFVP (h^−1^)	LFVP (h^−1^)	TVD (h^−1^)	HFVD (h^−1^)	LFVD (h^−1^)	VPR	BS	VMM (day^−1^)	CRL (μg l^−1^ day^−1^)
A	7.98 ± 2.33%	9.40 ± 1.68%	7.39 ± 2.34%	3.74 ± 0.98%	4.61 ± 0.83%	3.46 ± 1.06%	6.3 ± 0.5	35 ± 8	26.8 ± 2.1%	1.14 ± 0.16
B	12.48 ± 2.46%	27.95 ± 4.66%	11.98 ± 1.92%	0.80 ± 0.23%	2.81 ± 0.31%	0.62 ± 0.21%	1.6 ± 0.1	7 ± 2	70.5 ± 8.6%	8.62 ± 4.70
C	16.27 ± 2.85%	24.86 ± 3.29%	12.47 ± 4.58%	2.02 ± 0.33%	3.49 ± 0.56%	2.58 ± 0.50%	3.7 ± 0.3	28 ± 9	31.6 ± 3.5%	1.54 ± 0.31

*TVP* lytic total viral production rate, *HFVP* lytic high-fluorescence viral production rate, *LFVP* lytic low-fluorescence viral production rate, *TVD* total viral decay rate, *HFVD* high-fluorescence viral decay rate, *LFVD* low-fluorescence viral decay rate, *VPR* ratio of viral to prokaryotic abundance, *BS* burst size, *VMM* virus-mediated mortality of bacterioplankton, *CRL* C released by lysis

When the riverine and marine microbial communities were transferred into a simulated brackish water environment (i.e. FB + S and SB + F), the production of viruses was markedly reduced (Fig. 3). Previous studies have reported that viral production is tightly related to the metabolism of their hosts, which is controlled by nutritional parameters, such as DOC and inorganic nutrients [51, 52]. This may explain the reduction of the viral production in the FB + S treatment, in which the high DOC and nutrient concentrations were reduced via dilution with oligotrophic seawater, but it cannot explain the reduction in the SB + F treatment, in which the marine microbial hosts should have been provided more DOC and nutrients from the eutrophic waters. One possible explanation is that the carbon source and nutrient availability are not limiting factors for microbial activity in the brackish environment such that a “stimulation” effect of organic and inorganic nutrients on viral production was not observed in the SB + F treatment. Environmental factors other than nutrients may regulate microbial activity and therefore the productivity of viral particles. Microbes are sensitive to changes in ionic strength, and an increase in salinity can weaken the physiological state of microbiological cells [31, 53]. Studies have shown that seawater appears to have a strong inhibitory effect on riverine bacteria [54–56], whereas marine bacteria show high adaptability to variation in salinity [57]. In previous studies, seawater cause physiological stress on host metabolic processes [20, 54–56], which could include viral production, for infected cells, resulting in a decline of viral production. This mechanism can explain the stronger inhibition of viral production when the riverine microbes were transferred into seawater than when the marine microbes were transferred into freshwater. In addition, temperature is an important factor affecting viral production. In the cross-transplant experiment, the riverine and marine microbial communities were incubated at the in situ temperature of the brackish water environment, which was lower than the temperatures of their original environments (Table S1). Even a slight change of temperature can impact viral production through its regulation of the metabolic activity of the host microorganism [20]. Therefore, the large change in salinity and the decline in temperature might explain the inhibited viral production observed in both cross-transplant treatments. However, in order to elucidate the impact of individual parameter such as temperature or salinity on viral production and decay, further incubation experiments in field (e.g. with changing temperature but fixed salinity) and laboratory (based on isolated freshwater and marine phage-host system) are needed.

Our result showed the viral decay rates (total, high-, and low-fluorescence viruses) were inhibited when the riverine and marine viruses were transferred into simulated brackish water (Fig. 4). Compared to viral production, the decay of viral particles is considered to be more directly related to environmental factors [20]. Previous studies have shown that temperature is one of the most important factors affecting viral decay rate; it does so by directly damaging the biomolecular elasticity and molecular structure of proteins, such as membrane lipids and the protein capsids of viral particles [20]. For example, a positive correlation was obtained between the decay rate of *Escherichia coli* virus and temperature [18]. We previously found that warming significantly enhances the decay rates of viral communities in the western Pacific Ocean [21]. Therefore, in the experiment of freshwater–seawater cross-transplants, the inhibition of viral decay rate is partly explained by the lower in situ temperature at station B. Furthermore, changes in environmental factors (e.g. salinity and temperature) may decrease extracellular enzyme activity, which is another potential factor contributing to the inhibition. Extracellular enzymes (e.g. proteases and nucleases) are important factors affecting the viral decay rate by degrading viral protein capsids and the nucleic acids within [19, 22, 25]. Several studies have reported that the decay rate of viral particles is impacted by heat-labile substances, e.g. extracellular enzymes [15, 25, 58]. Low temperature may reduce enzyme activity through interference with the affinity of enzyme systems [59, 60]. In addition, a change of ionic strength may also lead to the inhibition of extracellular enzyme activity. For example, the different extracellular enzymes have specific adaptations to salinity, such as β-glucosidase and proteases [61, 62]. Thus, the decline of temperature and change of salinity in the treatments might have inhibited extracellular enzyme activity and resulted in the observed low viral decay rates. In addition, to maintain structural stability, bacteriophages appear to have specific ionic requirements, e.g. both Na^+^ and Mg^2+^ are necessary for bacteriophages to maintain the activity and stability to infect their hosts [63, 64]. Thus, changes in ionic strength might also have been important in accelerating the viral decay rate in the freshwater–seawater cross-transplants.

### High- vs. Low-Fluorescence Viruses

When members of the marine microbial community were transferred into virus-free freshwater (SB + F), pronounced differences in the production of high- and low-fluorescence viruses were observed. Based on the production rates observed at station C, the inhibition of high-fluorescence virus production (89.7%) was found to be stronger than that of low-fluorescence virus production (26.8%) (Fig. 3), suggesting that the marine hosts of low-fluorescence viruses may be more adaptable to changes in environmental conditions than those of high-fluorescence viruses. A metagenomic analysis of flow-cytometry-sorted marine virioplankton suggested that the high-fluorescence viruses (having high nucleic acid content) were dominated by eukaryotic algal viruses, whereas the low-fluorescence viruses (having low nucleic acid content) were dominated by bacteriophages [65]. Therefore, the different effects of freshwater–seawater mixing on production between high- and low-fluorescence viruses suggest that marine prokaryotic bacteria may be more adaptable to changes in environmental condition (e.g. DOC, nutrients and salinity) than are marine algae.

Similarly, regardless of whether members of the riverine viral community or marine viral community were transferred into brackish-like water (FV + S and SV + F), the effects on decay rate were markedly different between the high- and low-fluorescence viruses. The inhibition of high-fluorescence viral decay rate was stronger than that of low-fluorescence viral decay rate (80.5% and 46.6% vs. 12.8% and 21.1%). Based on the above discussion, this result suggests that algal viruses have more resistance to decay than do bacteriophages when transferred into simulated brackish water. Previous studies have shown that the viral decay rate increases significantly with the density of the packaged nucleic acid material of viruses [18]. Compared to bacteriophages, algal viruses typically have a lower density of packaged nucleic acid material [18, 21]. Therefore, when encountering rapid environmental changes such as a change in osmotic pressure, the capsid of bacteriophages seems to be more easily destroyed by internal pressure caused by the bending of nucleic acids and the strong repulsion between the highly charged neighbouring nucleic acid segments [66, 67]. This mechanism can explain why the nonindigenous low-fluorescence viruses showed higher decay rates than did the indigenous ones, with the bacteriophages being more prone to decay following a change of salinity. However, in contrast, the nonindigenous high-fluorescence virus decay rates were lower than indigenous ones, suggesting the algal viruses may be more prone to decay due to extracellular enzymes than to changes in salinity.

### Viruses in Brackish Waters

The freshwater–seawater cross-transplants showed that in situ viral production and decay at station B were different than they were under the FB/V + S and SB/V + F treatments. This result suggests that the mixing of nonindigenous hosts and viruses between the upstream river and downstream sea alone cannot veritably explain the in situ viral dynamics in brackish waters. Therefore, we hypothesised that brackish waters of PRE may harbour indigenous hosts and their viruses, which were supported by molecular ecology studies in other estuarine systems [68, 69]. In addition, compared with the in situ viral production and decay rates in brackish water, the nonindigenous viral production values (i.e. riverine and marine production values) were lower, but the nonindigenous viral decay rates were higher. These findings suggested a higher production rate and a lower decay rate of indigenous viruses of brackish water. However, this needs more experiments using the isolated viruses in laboratory or virus communities in natural environments to confirm. In addition, VMM ($=\frac{\Delta \mathrm{PA}}{{\mathrm{PA}}_0}$) and CRL (= ΔPA × 12.4 fg) are directly related to ΔPA during incubation period, which may be overestimated since some nonindigenous prokaryotic bacteria are not suitable to survive in brackish water environment, resulting in death. But they were significantly higher in the brackish water than in the freshwater and seawater. This may indicate that viruses are important in mediating the carbon cycle and biogeochemical cycles via the viral shunt in brackish water areas.

An interesting observation is that the lowest viral population size (abundance) together with high in situ viral production and the lowest in situ viral decay rate were observed in brackish water. This finding indicated the possible overestimation of viral production or possible underestimation of viral decay from the in situ measurements. According to the experimental design used for viral production measurement, the estimation considered viral particles produced on particles less than 3 μm in diameter (as the water samples were pre-filtered with 3-μm filters). However, due to the 0.22-μm filtration, the estimation of viral decay excluded all particles that can adsorb viral particles and protozoa that can passively graze them. Station B, in the brackish water area, receives large amounts of organic and inorganic particles, which may have caused the overestimation of viral production or the underestimation of viral decay. In addition, the adsorption of large numbers of viruses by organic and inorganic particles in the brackish water area may be an important route by which the carbon and other elements of viral particles sink into the sediment.

## Conclusions

As important members of marine ecosystems, viruses affect the microbial ecology and biogeochemical cycles of the marine environment by releasing DOC and nutrients into the environment by host lysis and the decay of viral particles. Our study showed complex viral ecological dynamics in a typical estuary area based on in situ observations and a freshwater–seawater cross-transplant experiment. The results showed that the mixing of freshwater and seawater caused decreases in viral production and decay rates, indicating that changes in environmental factors, such as temperature, salinity, DOC and nutrients, due to freshwater–seawater mixing impact the ecological behaviours of virioplankton in the estuary. Furthermore, the high- and low-fluorescence viruses showed different responses to water mixing. Together, these findings improve our knowledge of the ecological dynamics of viruses in the estuarine environment and our understanding of microbial ecology in complex estuary ecosystems.

## Electronic Supplementary Material

ESM 1 (DOCX 4508 kb)
