# Supplementary material for: Impacts of Freshwater and Seawater Mixing on the Production and Decay of Virioplankton in a Subtropical Estuary
Source: Microb Ecol. 2019 Apr 10;78(4):843–54. doi: 10.1007/s00248-019-01362-2 (PMC6842343; doi:10.1007/s00248-019-01362-2)
Supplement: Supplementary file 1 — (DOCX 4508 kb) [file 248_2019_1362_MOESM1_ESM.docx]

**Impacts of freshwater and seawater mixing on the production and decay of virioplankton in a subtropical estuary**

Wei Wei^1,2^, Nannan Wang^1,2^, Lanlan Cai^2,3^, Chuanlun Zhang^4^, Nianzhi Jiao^2,3^*, Rui Zhang^2,3^*

^1^College of the Environment and Ecology, Xiamen University, Xiamen 361102, PR China

^2^State Key Laboratory of Marine Environmental Science, Institute of Marine Microbes and Ecospheres, Xiamen University, Xiamen 361102, PR China

^3^College of Ocean and Earth Sciences, Xiamen University, Xiamen 361102, PR China

^4^Department of Ocean Science and Engineering, Southern University of Sciences and Technology, Shenzhen 518055, PR China

*Corresponding authors: Rui Zhang (ruizhang@xmu.edu.cn); Nianzhi Jiao (jiao@xmu.edu.cn)


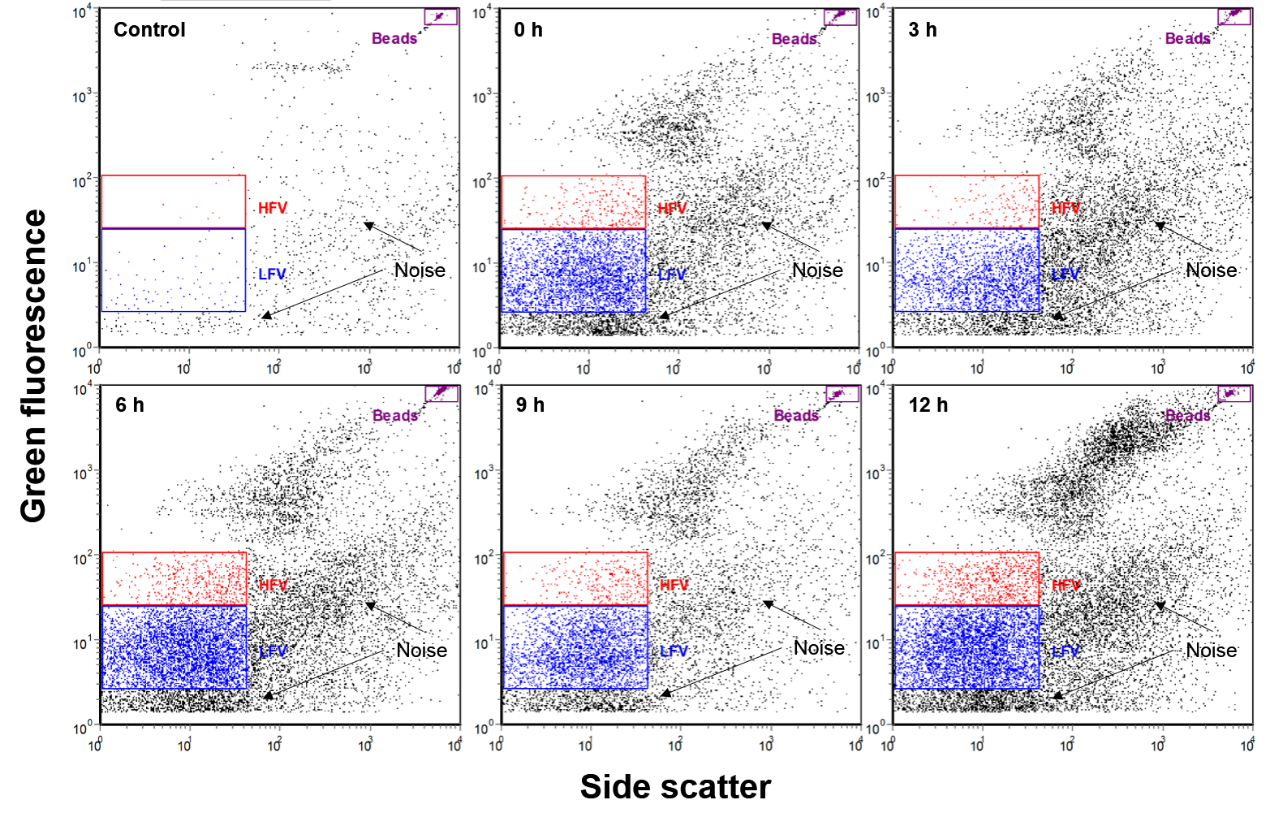


**FIG S1 The scatter diagrams of flow cytometry analysis for high- and low-fluorescence viruses during the viral production experiment (100% of particles plotted). The control was 0.02 µm-filtered sample water. The beads were 1 µm in diameter with yellow-green fluorescence (Molecular Probes). HFV: high-fluorescence viruses; LFV: low-fluorescence viruses**


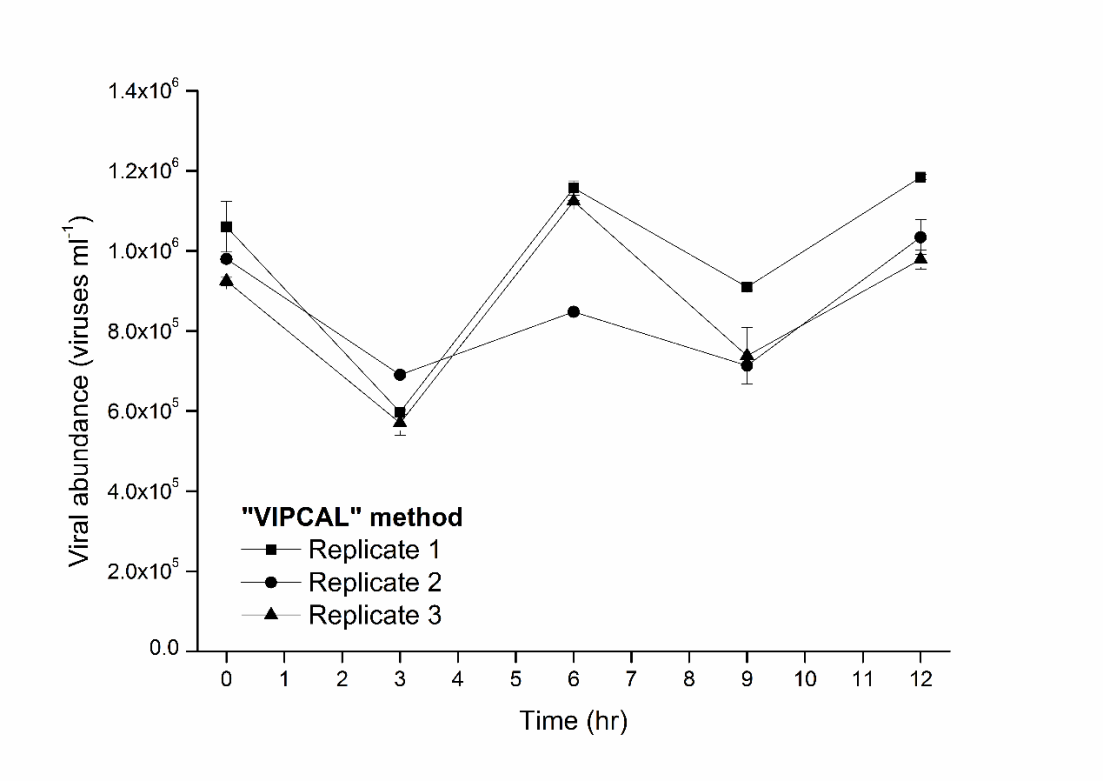


**FIG S2 The plot of VIPCAL method for the viral abundance vs. time during 0 to 12 h in total viral production experiment of Station B from triplicate incubations. The error bars were calculated by the two technical repetition of each sample**

**
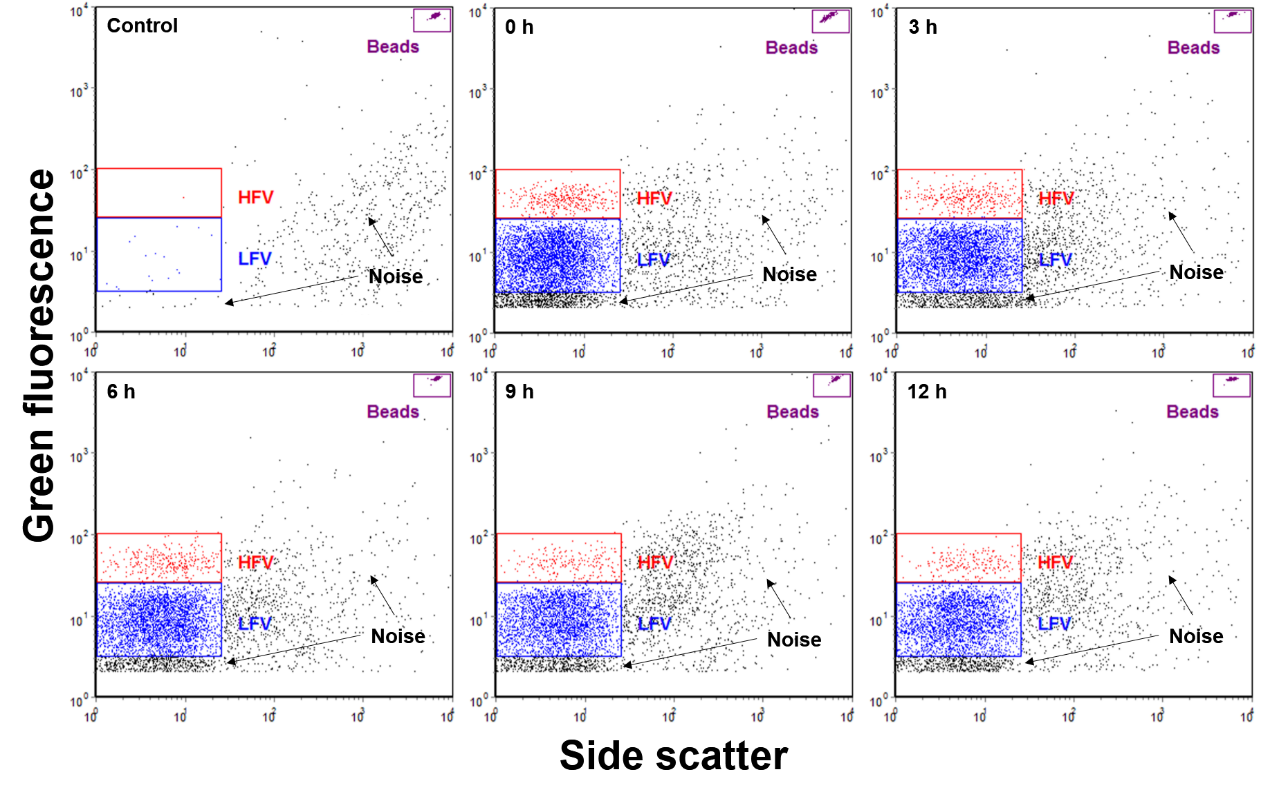
**

**FIG S3 The scatter diagrams of flow cytometry analysis for high- and low-fluorescence viruses during the viral decay experiment (50% of particles plotted). The control was 0.02 µm-filtered sample water. The beads were 1 µm in diameter with yellow-green fluorescence (Molecular Probes). HFV: high-fluorescence viruses; LFV: low-fluorescence viruses**


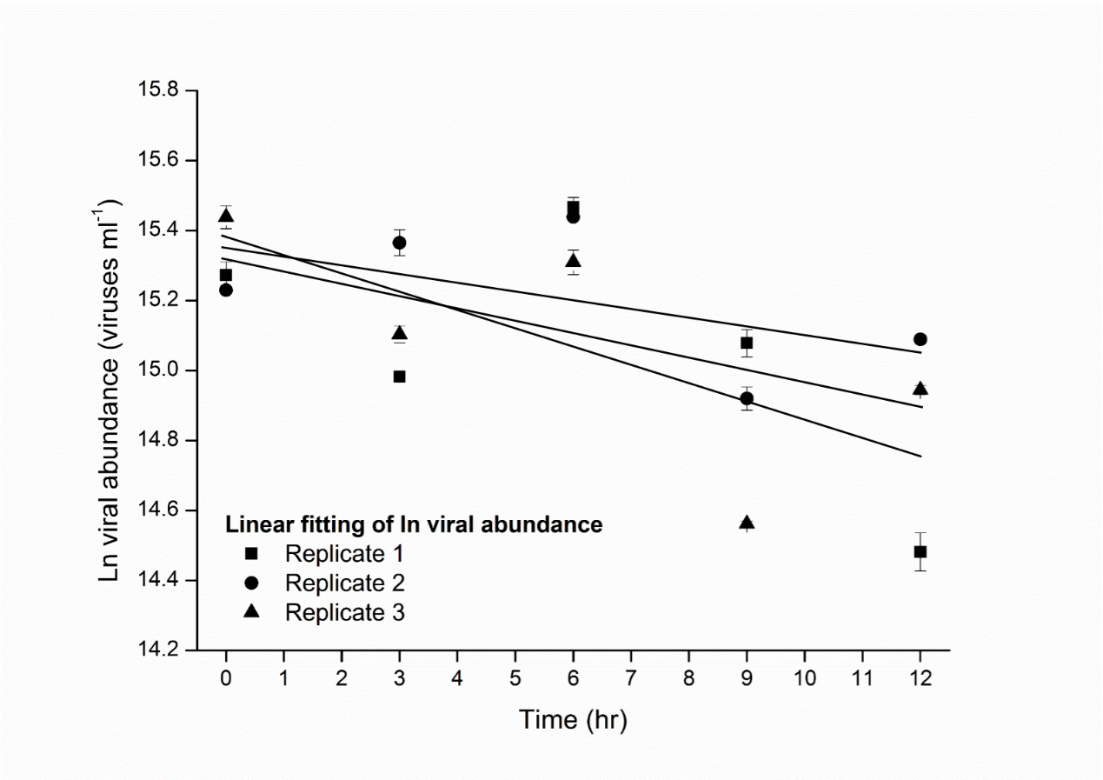


**FIG S4 The plot of linear regression of the ln viral abundance vs. time during 0 to 12 h in low-fluorescence viral decay experiment of Station A from triplicate incubations. The error bars were calculated by the two technical repetition of each sample**

**
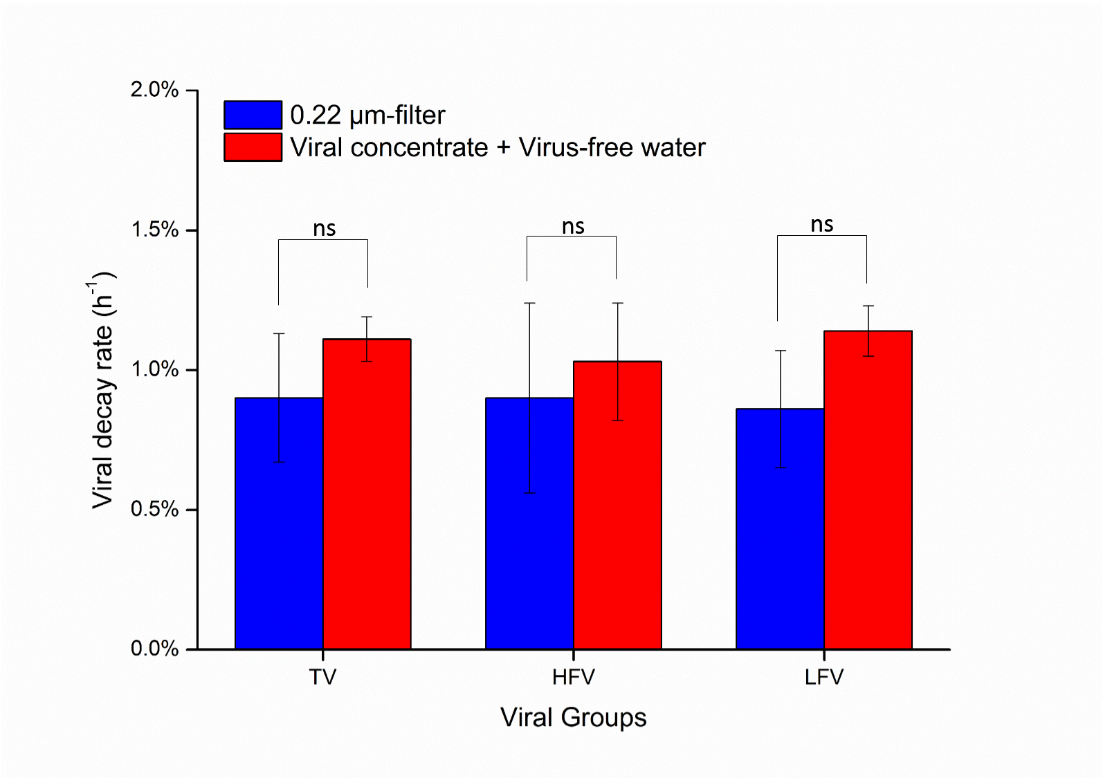
**

**FIG S5 T-test analysis of significant differences among total, high-, and low-fluorescence virus decay rates by the treatment of 0.22 µm-filter and viral concentrate + virus-free water. Error bars indicate the standard deviations calculated from triplicate sample measurements. TV: total viruses; HFV: high-fluorescence viruses; LFV: low-fluorescence viruses; ns: no significant difference.**


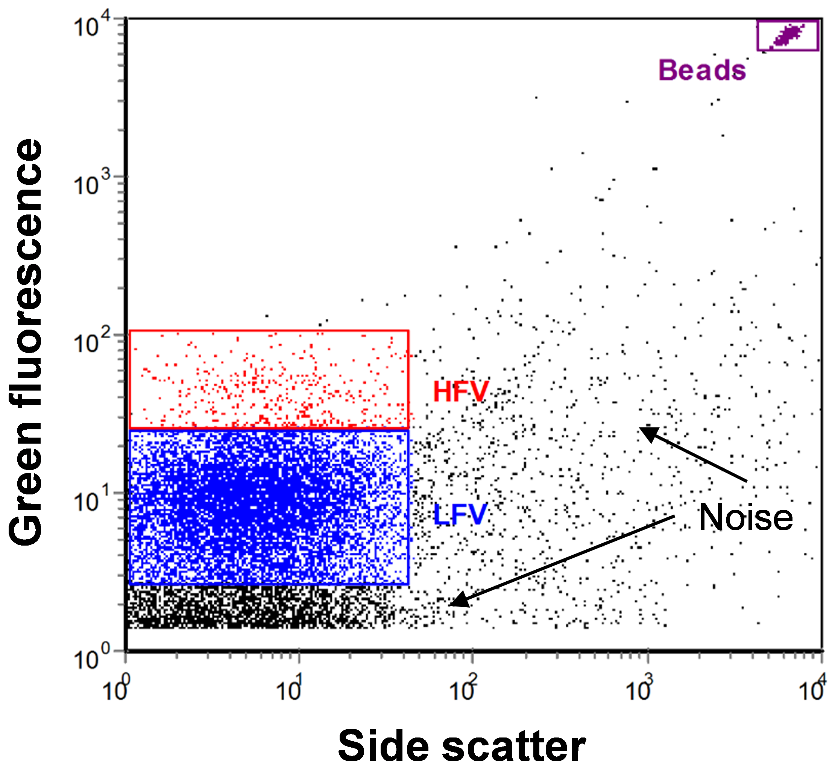


**FIG S6 Scatter diagram of high- and low-fluorescence viruses detected by flow cytometry (100% of particles plotted). The beads were 1 µm in diameter with yellow-green fluorescence (Molecular Probes). HFV: high-fluorescence viruses; LFV: low-fluorescence viruses**

| **TABLE S1 Physicochemical parameters of the three stations in Pearl River Estuary.** | | | | | | | | |
| --- | --- | --- | --- | --- | --- | --- | --- | --- |
| Station | Latitude | Longitude | Temperature | Salinity | Turbidity | pH | DO | ORP |
|  | (°N) | (°E) | (°C) |  | (FTU) |  | (mg l^–1^) | (mV) |
| A | 22.90 | 113.57 | 29.1 | 0.14 | 13 | 6.14 | 0.85 | 295 |
| B | 22.36 | 113.78 | 28.0 | 18.10 | 10 | 8.02 | 5.99 | 172 |
| C | 21.21 | 113.94 | 29.2 | 36.06 | 0 | 8.25 | 6.47 | 91 |
| **Abbreviations represent dissolved oxygen (DO) and oxidation-reduction potential (ORP).** | | | | | | | | |
